# Association between interleukin-2 cytokine levels and *Plasmodium* infections: a systematic review and meta-analysis

**DOI:** 10.1186/s12879-025-11977-1

**Published:** 2025-11-05

**Authors:** Pattamaporn Kwankaew, Kwuntida Uthaisar Kotepui, Nsoh Godwin Anabire, Polrat Wilairatana, Manas Kotepui

**Affiliations:** 1 Medical Technology, School of Allied Health Sciences, Walailak University, Tha Sala, Nakhon Si Thammarat, Thailand; 2 Medical Technology, Faculty of Science, Nakhon Phanom University, Nakhon Phanom, Thailand; 3 Department of Biochemistry & Molecular Medicine, School of Medicine, University for Development studies, Tamale, Ghana; 4 West African Centre for Cell Biology of Infectious Pathogens (WACCBIP), Department of Biochemistry, Cell & Molecular Biology, University of Ghana, Accra, Ghana; 5 Department of Clinical Tropical Medicine, Faculty of Tropical Medicine, Mahidol University, Bangkok, Thailand

**Keywords:** Interleukin-2, IL-2, Malaria, *Plasmodium*, Cytokines, Systematic review

## Abstract

**Background:**

Interleukin-2 (IL-2) is a central cytokine in T-cell mediated immunity, playing a dual role in both pro-inflammatory responses and immune regulation. While cytokines such as IL-6 and tumor necrosis factor-α (TNF-α) have been extensively studied in malaria pathogenesis, the role of IL-2 remains poorly understood and inconsistently reported across studies. This systematic review and meta-analysis aimed to synthesize available evidence on IL-2 levels in malaria patients and assess their association with disease severity.

**Methods:**

A systematic search was conducted across five databases (PubMed, MEDLINE, EMBASE, Scopus, and CENTRAL) without date restrictions. Studies were eligible if they reported IL-2 levels in human participants with malaria, compared to uninfected individuals, and/or across malaria severity. Risk of bias was assessed using Joanna Briggs Institute (JBI) tools. Standardized mean differences (SMD) were calculated using a random-effects model. Heterogeneity, subgroup analyses, meta-regression, and publication bias were evaluated using established statistical methods.

**Results:**

Out of 3,023 records screened, 30 studies met the inclusion criteria for the systematic review. Most studies reported no significant differences in IL-2 levels between individuals with malaria and uninfected controls. The meta-analysis confirmed this finding, showing no significant difference (*P* = 0.25, SMD = 4.56, 95% CI [-3.16; 12.29], *I²* = 98.6%, 1074 participants, random-effects model). Similarly, the majority of studies comparing IL-2 levels between severe and non-severe malaria cases found no significant differences. Meta-analysis results were consistent, showing no significant association (*P* = 0.57, SMD = 0.37, 95% CI [-0.91; 1.67], *I²* = 97.4%, 694 participants, random-effects model). Subgroup analyses suggested that geographic region significantly influenced IL-2 expression patterns.

**Conclusion:**

This systematic review and meta-analysis found no consistent evidence of altered IL-2 levels in individuals with *Plasmodium* infection compared to uninfected controls, nor between patients with severe and non-severe malaria. However, substantial heterogeneity across studies limits the interpretability of these findings. Future well-designed studies that account for geographic, methodological, and host-related factors are needed to determine whether IL-2—alone or in combination with other immunological markers—can serve as a reliable biomarker for malaria infection or disease severity.

**Supplementary Information:**

The online version contains supplementary material available at 10.1186/s12879-025-11977-1.

## Introduction

Malaria is a parasitic disease caused by protozoan parasites from the genus *Plasmodium*, transmitted to humans through the bites of infected female *Anopheles* mosquitoes [1]. Although over 172 species of *Plasmodium* are recognized, only five species cause malaria in humans: *P. falciparum*, *P. malariae*, *P. vivax*, *P. ovale*, and *P. knowlesi* [1]. Malaria remains a major global health concern, particularly in tropical regions [2–4]. The disease can escalate rapidly, leading to severe complications such as anemia, organ failure, and cerebral malaria, with potentially fatal outcomes, especially among young children and immunocompromised individuals [5]. The pathogenesis of malaria is influenced by various factors, including the host immune response, parasite strain variations, and co-infections, all of which present complex challenges for disease progression and treatment [6].

The immune response to malaria is complex and involves the interplay of multiple cytokines, immune cells, and pathways [7]. In malaria, cytokines play crucial roles in regulating the balance between pro-inflammatory responses to control the parasite and anti-inflammatory responses to limit tissue damage [8]. Distinct cytokine levels offer insights into disease progression across different malaria stages, as highlighted by comprehensive reviews and meta-analyses on innate immune cytokines and malaria severity. A previous systematic review and meta-analysis showed that elevated interleukin (IL)-8 levels are consistently observed in malaria patients compared to uninfected individuals. However, its association with disease severity remains unclear, as no significant differences in IL-8 levels have been found between patients with severe and non-severe malaria [9]. Similarly, the review demonstrated that elevated IL-6 levels are associated with severe malaria, suggesting its potential as a marker of disease severity [10]. Additionally, higher levels of IL-1β and tumor necrosis factor (TNF) -α have been linked to malaria severity, underscoring their importance in the inflammatory response [11, 12]. In terms of anti-inflammatory cytokines, the review revealed that decreased transforming growth factor (TGF)-β levels are seen in patients with uncomplicated malaria compared to healthy controls, while IL-10 levels are elevated in severe malaria, suggesting their roles in regulating inflammation and immune responses during malaria infection [13, 14].

Interleukin-2, a classic four-helix globular protein with an approximate molecular weight of 15.5-kDa, stands prominent [15]. This cytokine is typically produced by cluster of differentiation (CD) 4 + T lymphocytes in secondary lymphoid organs; these organs house naive, memory, and T helper (Th) 1 cells that respond to antigenic stimulation [16–20]. Additionally, IL-2 production has been noted in a range of other cells, including naive CD8 + T cells, dendritic cells, thymic cells, natural killer (NK) cells, natural killer T (NKT) cells, and mast cells [21, 22]. The cytokine can undergo various glycosylation processes, resulting in attached carbohydrate molecules, a modification that can influence both its stability and activity [23]. IL-2 is a key cytokine involved in the activation and proliferation of T-cells, which are central to the adaptive immune response against pathogens including malaria parasites. In severe malaria, dysregulated T-cell responses may contribute to immunopathology. Elevated or suppressed IL-2 levels may indicate abnormal T cell activation or dysfunction associated with severe disease [24].

In this study, IL-2 was selected for investigation due to its unique role in modulating both pro-inflammatory and regulatory immune responses, which sets it apart from the other cytokines examined. While cytokines such as IL-6, IL-1β, and TNF-α have been extensively studied in relation to malaria severity, the role of IL-2 in immune regulation, T cell activation, and its potential dual contributions to both protective and pathogenic responses remain less well understood. Moreover, findings on the role of IL-2 in malaria patients have been inconsistent. For instance, some studies have demonstrated significantly increased IL-2 levels in patients with malaria compared to uninfected individuals [25, 26], while others have found no significant difference between the two groups [27, 28], or even decreased IL-2 levels in malaria patients [29, 30]. As a result, it is imperative to establish a more comprehensive understanding of IL-2’s function in varying clinical outcomes associated with malaria. This systematic review and meta-analysis aim to address this gap by examining the differences in IL-2 levels among patients with differing malaria severity, including uncomplicated cases, compared to a control group of healthy individuals. Insights gained from this study may help guide the development of diagnostic methods or therapeutic strategies aimed at modulating IL-2 levels to improve patient outcomes in malaria.

## Methods

### Registration of protocol and reporting guideline

The systematic review protocol was registered in the International Prospective Register of Systematic Reviews (PROSPERO) under the ID: CRD42022365486. The results of the systematic review and meta-analysis were reported in accordance with the Preferred Reporting Items for Systematic reviews and Meta-Analyses (PRISMA) guidelines [31].

### Search strategy

A comprehensive literature search was conducted across several databases, including EMBASE, Scopus, MEDLINE, PubMed, and Cochrane Central Register of Controlled Trials (CENTRAL). The search strategy was constructed using the keywords ‘malaria’ and ‘Interleukin-2,’ formatted as follows: ‘(“Interleukin 2” OR IL-2 OR IL2 OR TCGF OR “Interleukin 2” OR “Lymphocyte Mitogenic Factor” OR “T-Cell Growth Factor” OR “T Cell Growth Factor” OR “T-Cell Stimulating Factor” OR “T Cell Stimulating Factor” OR “Thymocyte Stimulating Factor” OR “Interleukin II”) AND (malaria OR *Plasmodium* OR “remittent fever” OR “marsh fever” OR paludism).’ Details of the search strategy in each database are shown in Table S1. There were no limitations on the publication date of the articles.

### Eligibility criteria

Studies had to meet specific criteria: they must qualitatively or quantitatively measure IL-2 levels in human participants, compare IL-2 levels between individuals with malaria and uninfected controls, and optionally provide data comparing IL-2 levels between cases of severe and non-severe malaria. Furthermore, only studies that were peer-reviewed and published in scientific journals were considered for inclusion. Exclusions were made for studies involving animal models, in vitro research, case report, letter, review, and conference abstracts. Additionally, studies that investigated IL-2 in co-infections or only reported IL-2 mRNA expression levels were also excluded.

### Study selection and data extraction

After duplicate records were removed, the remaining studies underwent preliminary screening based on their titles and abstracts. This screening was performed by two independent authors, and any disagreements were resolved through consensus. Subsequently, the remaining studies were assessed in full text against the eligibility criteria, with reasons for exclusion clearly documented. Two authors (PK, MK) independently extracted the following data from each eligible study: first author, year of publication, study design, country, number of participants, mean and standard deviation (or median and range) of IL-2 levels for each group, diagnostic method for malaria, type of blood sample used for IL-2 measurement, and the method used for IL-2 measurement (Table S2). Any discrepancies in data extraction were resolved through discussion or by involving a third reviewer.

### Quality assessment

The Joanna Briggs Institute (JBI) Critical Appraisal tools provide checklists for evaluating the quality and risk of bias in various types of studies, including cohort, case-control, and cross-sectional studies [32]. Common criteria across these checklists focus on the clarity of research objectives, the validity and reliability of exposure and outcome measurements, and the handling of confounding factors. For cohort studies, additional emphasis is placed on the comparability of the exposed and unexposed groups and adequacy of follow-up. Case-control studies require careful selection and description of cases and controls, while cross-sectional studies necessitate clear criteria for sample inclusion. Ethical considerations are a common element in all checklists. Typically, each criterion is assessed with a “Yes,” “No,” or “Unclear” response, accompanied by comments or justifications from the appraiser.

### Definition of outcomes

Severe falciparum malaria is defined by the presence of one or more of the following complications in conjunction with *P. falciparum* asexual parasitemia: impaired consciousness, prostration, multiple convulsions, acidosis, hypoglycemia, severe malarial anemia, renal impairment, jaundice, pulmonary edema, significant bleeding, shock, and hyperparasitemia [33]. Severe vivax malaria follows the same criteria as falciparum malaria, but without specific parasite density thresholds. Non-severe malaria is characterized by the absence of the complications listed in the WHO criteria for severe malaria, while still presenting with malaria parasitemia [33].

### Data analysis

A qualitative synthesis using thematic analysis was conducted to determine the findings regarding the trend of IL-2 alterations between patients with malaria and uninfected individuals, as well as between patients with severe and non-severe malaria [34]. A quantitative synthesis using meta-analysis was performed with a random-effects model, accounting for the anticipated high heterogeneity among the included studies [35]. Standardized mean differences (SMDs) and their 95% confidence intervals (CIs) were calculated as the effect size measure for comparing IL-2 levels between different groups. This approach involves standardizing the mean differences in IL-2 levels, thereby enabling a more comparable combination of data across studies [36]. The *I*^*2*^ statistic was used to assess heterogeneity among the studies, with *I*^*2*^ values of 25%, 50%, and 75% considered as indicators of low, moderate, and high heterogeneity, respectively [37]. Two primary analyses were conducted: one examining the difference in IL-2 levels between patients with malaria and uninfected individuals, and the other evaluating the difference in IL-2 levels between patients with severe and non-severe malaria. Meta-regression and subgroup analyses were conducted based on publication year, study design, continent, age group, *Plasmodium* species, malaria diagnostic method, IL-2 measurement method, and blood sample type to identify confounding factors that might affect the pooled effect estimate. Tests for publication bias, including visualization of funnel plot asymmetry and Egger’s test for small-study effects, were performed when sufficient studies (≥ 10) were available [38]. The influential analysis, which omitting one study at a time, was applied to demonstrate the stability of the meta-analysis results [39]. All statistical analyses were performed using RStudio (Version: 2024.04.2 + 764) [40].

## Results

### Search results

**Fig. 1:**
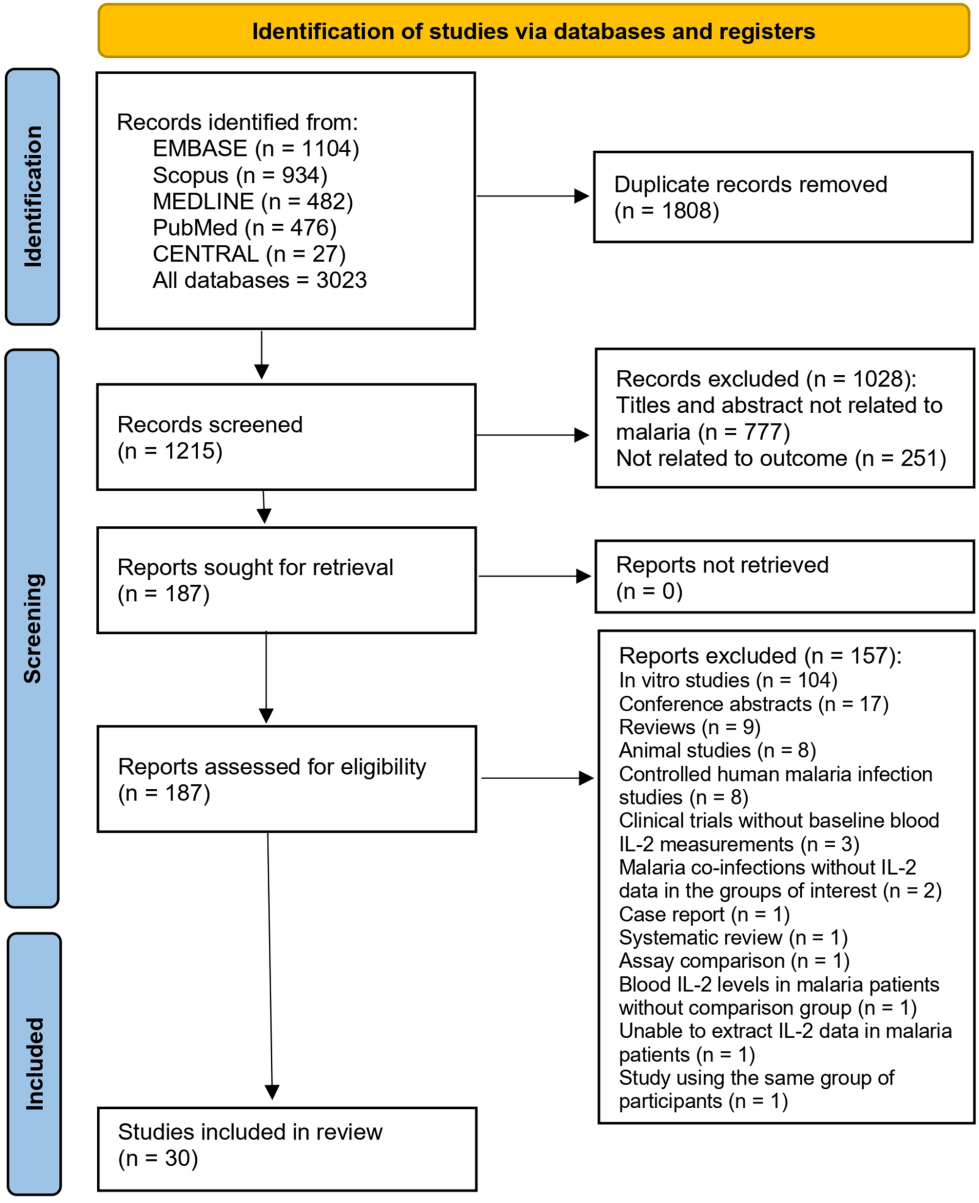
Study flow diagram

A total of 3,023 records were initially identified through various databases: EMBASE (*n* = 1104), Scopus (*n* = 934), MEDLINE (*n* = 482), PubMed (*n* = 476), and CENTRAL (*n* = 27). After removing 1,808 duplicate records, 1,215 records were screened for relevance. Of these, 1028 records were excluded for the following reasons: titles and abstracts not related to malaria (*n* = 777), and not related to the intended outcome (*n* = 251). Out of the 187 reports assessed for eligibility, 157 were excluded for various reasons: in vitro studies (*n* = 104), conference abstracts (*n* = 17), reviews (*n* = 9), animal studies (*n* = 8), controlled human malaria infection studies (*n* = 8), clinical trials without baseline blood IL-2 measurements (*n* = 3), and other reasons (*n* = 8). Consequently, 30 studies [25–30, 41–64] were included in the review (Fig. 1).

### Characteristics of included studies

The characteristics of the 30 studies included in the systematic review are summarized in Table 1. The publication years of the studies in this systematic review range from 2000 to 2024, with the most frequent publications occurring between 2010 and 2019 (*n* = 15, 50%). Regarding study designs, the majority were case-control studies (*n* = 14, 46.67%), followed by cross-sectional (*n* = 12, 40%) and cohort studies (*n* = 4, 13.33%). The geographical focus of these studies spanned four continents, with most conducted in Africa (*n* = 15, 50%), followed by South America (*n* = 9, 30%), Asia (*n* = 5, 16.67%), and one in Europe (France). The majority of studies (*n* = 21, 70%) investigated *P. falciparum*, while the remaining studies (*n* = 9, 30%) focused on *P. vivax*. Most studies (*n* = 13, 43.33%) covered various age ranges, followed by those focusing on children (*n* = 8, 26.67%) and adults (*n* = 3, 10%). In terms of malaria detection methods, the majority (*n* = 21, 70%) used microscopy, followed by Microscopy/PCR (*n* = 5, 16.67%). For IL-2 quantification, most studies (*n* = 18, 60%) employed bead-based assays, followed by ELISA (*n* = 9, 30%). Finally, the majority used plasma (*n* = 16, 53.33%) or serum (*n* = 13, 43.33%) for sample analysis. The detailed characteristics of each individual study included in the systematic review are shown in Table S2.

**Table 1:** Summary characteristics of included studies

Characteristics	Total 30 studies	%
**Publication year**		
2020–2024	7	23.33
2010–2019	15	50.00
2000–2009	6	20.00
Before 2000	2	6.67
**Study designs**		
Case-control study	14	46.67
Cross-sectional study	13	43.33
Cohort study	3	10.00
**Study areas**		
Africa	15	50.00
Ghana	5	16.67
Nigeria	4	13.33
Kenya	1	3.33
Mozambique	1	3.33
Sudan	1	3.33
Malawi	1	3.33
Democratic Republic of São Tomé and Príncipe	1	3.33
Cameroon	1	3.33
South America	9	30.00
Brazil	8	26.67
Columbia	1	3.33
Asia	5	16.67
India	3	10.00
Sri Lanka	1	3.33
Turkey	1	3.33
Europe (France)	1	3.33
***Plasmodium*** **spp.**		
* P. falciparum*	21	70.0
* P. vivax*	9	30.0
**Participants**		
Children	8	26.67
Adults	3	10.0
Pregnant women	2	6.67
Children and adults	13	43.33
Not specified	4	13.33
**Methods for malaria detection**		
Microscopy	21	70.0
RDT	1	3.33
Microscopy/ RDT	2	6.67
Microscopy/PCR	5	16.67
Microscopy/RDT/ PCR	1	3.33
**Methods for IL-2 quantification**		
Bead-based assay	18	60.0
ELISA	9	30.0
ELISA/EIA	2	6.67
RIA	1	3.33
**Blood samples for IL-2 measurement**		
Plasma	16	53.33
Serum	13	43.33
Plasma/serum	1	3.33

Abbreviations: EIA, Enzyme immunoassay; ELISA, Enzyme-linked immunosorbent assay; PCR, Polymerase chain reaction; RDT, Rapid diagnostic test; RIA, radioimmunoassay

### Quality of the included studies

The results of the JBI critical appraisal checklist applied to several analytical cross-sectional studies [25–28, 42, 44, 49, 50, 52, 57, 58, 61, 63] indicated that most studies met the majority of the inclusion criteria. However, some studies did not adequately identify or address confounding factors [25, 27, 28]. The JBI critical appraisal checklist for case-control studies revealed that all evaluated studies sufficiently met the majority of the criteria [29, 30, 43, 45, 47, 51, 53–56, 59, 60, 62, 64]. While most studies-maintained standardization in measuring exposure and assessing outcomes for both cases and controls, a common shortfall was the failure to identify and implement strategies to address confounding factors [29, 30, 45, 47, 53, 59, 60, 64]. For cohort studies [41, 46, 48], the majority of the criteria outlined in the checklist were successfully met (Table S3).

### Qualitative and quantitative syntheses of IL-2 levels in malaria and uninfected individuals

Among the 23 studies that investigated IL-2 levels in both malaria patients and uninfected individuals, the majority (*n* = 12, 52.2%) reported no significant difference in IL-2 levels between the two groups [27, 28, 42, 44, 45, 47, 48, 55, 59, 62, 63, 65]. That notwithstanding, some studies (*n* = 8, 34.8%) found a significant increase in IL-2 levels in malaria patients compared to uninfected individuals [26, 43, 50, 54, 60, 61, 64, 66]. While only two studies (*n* = 2, 8.7%) reported a significant decrease in IL-2 levels in malaria patients compared to uninfected individuals [30, 67]. One study reported increased IL-2 levels in severe malaria compared to febrile controls but found no significant difference between uncomplicated malaria and febrile controls [51] (Table 2).

**Fig. 2:**
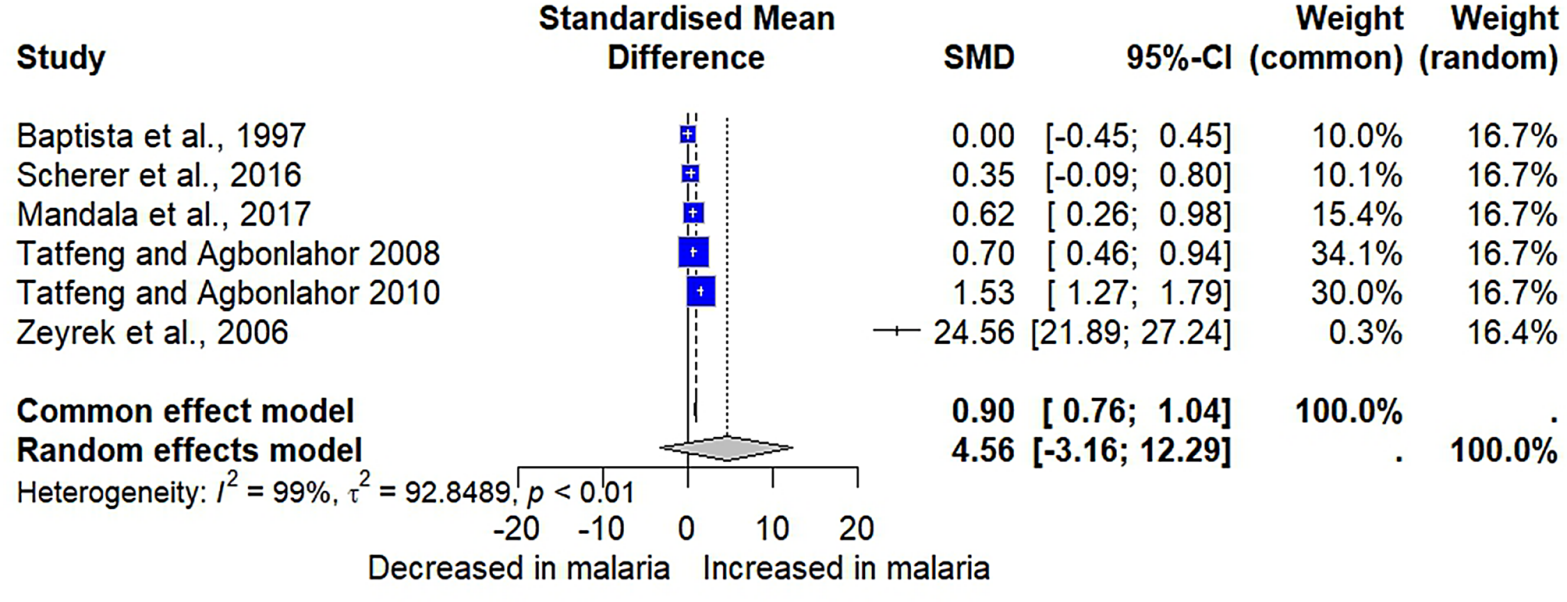
The forest plot compared IL-2 levels in patients with malaria compared to uninfected individuals. Explanations of the forest plot; blue square, effect estimate of individual study; gray diamond, pooled effect estimate; dash vertical line, pooled effect estimate; gray vertical line, no effect line; CI, confidence interval. SMD, standardized mean difference

Six studies provided data for a meta-analysis comparing IL-2 levels between patients with malaria (*n* = 629) and uninfected individuals (*n* = 445) [26, 28, 57, 59, 60, 64]. The results showed no significant difference in IL-2 levels between patients with malaria and uninfected individuals (*P* = 0.25, SMD = 4.5642, 95% CI [-3.1598; 12.2881], *I²* = 98.6%, 1074 participants, random-effects model, Fig. 2). However, the fixed-effects model showed a significant increase in IL-2 levels in patients with malaria compared to uninfected individuals (*P* < 0.0001, SMD = 0.8998, 95% CI [0.7578; 1.0417], Fig. 2).

**Table 2:** Comparison of IL-2 levels between groups of patients with malaria and uninfected individuals, severe and non-severe malaria patients

No.	Authors	Continents	Country	*Plasmodium* spp.	Age groups	Comparison of IL-2 levels between groups of patients with malaria and uninfected individuals
1.	Acero et al., 2022 [41]	South America	Colombia	*P. vivax*	Children and adults	No statistically significant differences in IL-2 between severe malaria and non-severe malaria patients.
2.	Anabire et al., 2019 [42]	Africa	Ghana	*P. falciparum*	Pregnant women	No statistically significant differences in IL-2 between malaria and uninfected individuals.
3.	Armah et al., 2007 [27]	Africa	Ghana	*P. falciparum*	Children	No statistically significant differences in IL-2 between cerebral malaria, severe malarial anemia, and non-malaria.
4.	Baptista et al., 1997 [28]	Africa	Democratic Republic of São Tomé and Príncipe	*P. falciparum*	Children	No statistically significant differences in IL-2 between cerebral malaria, mild malaria, and non-malaria.
5.	Bin Eric et al., 2024 [43]	Africa	Cameroon	*P. falciparum*	Children and adults	IL-2 levels were significantly increased in patients with malaria as compared to uninfected individuals.
6.	Chaves et al., 2016 [66]	South America	Brazil	*P. vivax*	Children and adults	IL-2 levels were significantly increased in patients with malaria as compared to uninfected individuals.
7.	Coelho et al., 2013 [44]	South America	Brazil	*P. vivax*	Not specified	No statistically significant differences in IL-2 between malaria and uninfected individuals.
8.	da Costa et al., 2014 [67]	South America	Brazil	*P. vivax*	Adults	IL-2 levels were significantly decreased in patients with malaria as compared to uninfected individuals.
9.	de Jesus et al., 2023 [45]	South America	Brazil	*P. vivax*	Children and adults	No statistically significant differences in IL-2 between patients with malaria patients and uninfected individuals.
10.	de Roquetaillade et al., 2023 [46]	Europe	France	*P. falciparum*	Adults	No statistically significant differences in IL-2 between severe malaria and uncomplicated malaria patients.
11.	Frimpong et al., 2022 [47]	Africa	Ghana	*P. falciparum*	Not specified	- IL-2 levels were significantly increased in clinical malaria as compared to non-malarial sepsis. - No difference in IL-2 levels between clinical malaria and febrile controls. - No difference in IL-2 levels between non-malarial sepsis and febrile controls.
12.	Jain et al., 2008 [48]	Asia	India	*P. falciparum*	Children and adults	No statistically significant differences in IL-2 between malaria (cerebral malaria non-survivors, cerebral malaria survivors, mild malaria) and healthy controls.
13.	Kremsner et al., 1990 [30]	South America	Brazil	*P. falciparum*	Children and adults	IL-2 levels were significantly decreased in patients with malaria as compared to uninfected individuals.
14.	Mandala et al., 2017 [26]	Africa	Malawi	*P. falciparum*	Children	- IL-2 levels were significantly increased in patients with cerebral malaria and severe malarial anemia as compared to uninfected individuals. - IL-2 levels were significantly higher in severe malaria (cerebral malaria and severe malarial anemia) as compared to uncomplicated malaria.
15.	Mendonça et al., 2015 [68]	South America	Brazil	*P. vivax*	Children and adults	- IL-2 levels were significantly increased in patients with severe malaria as compared to mild malaria.
16.	Menezes et al., 2018 [50]	South America	Brazil	*P. vivax*	Children and adults	IL-2 levels were significantly increased in patients with malaria compared to endemic controls.
17.	Obeng-Aboagye et al., 2023 [51]	Africa	Ghana	*P. falciparum*	Children	- IL-2 levels were significantly increased in patients with severe malaria compared to febrile controls. - No difference in IL-2 levels between patients with severe and uncomplicated malaria patients. - No difference in IL-2 levels between patients with uncomplicated malaria and febrile controls.
18.	Ong’echa et al., 2011 [52]	Africa	Kenya	*P. falciparum*	Children	No statistically significant differences in IL-2 between severe (non-severe malarial anemia and severe malarial anemia) and uncomplicated malaria patients.
19.	Perera et al., 2013 [53]	Asia	Sri Lanka	*P. falciparum*	Children and adults	IL-2 levels were not detectable in both severe and uncomplicated malaria (< 2 pg/ml).
20.	Prakash et al., 2006 [54]	Asia	India	*P. falciparum*	Children and adults	- IL-2 levels were significantly increased in malaria (severe, mild, cerebral malaria) as compared to uninfected individuals (endemic and non-endemic controls). - No difference in IL-2 levels between severe and mild malaria. - IL-2 levels were significantly decreased in cerebral malaria as compared to mild malaria patients.
21.	Rabiu et al., 2022 [55]	Africa	Nigeria	*P. falciparum*	Pregnant women	Second trimester: - No statistically significant differences in IL-2 between malaria and uninfected individuals. Third trimester: - No statistically significant differences in IL-2 between malaria and uninfected individuals.
22.	Rovira-Vallbona et al., 2012 [56]	Africa	Mozambique	*P. falciparum*	Children	No statistically significant differences in IL-2 between severe and uncomplicated malaria.
23.	Scherer et al., 2016 [65]	South America	Brazil	*P. vivax*	Not specified	No statistically significant differences in IL-2 between malaria and uninfected individuals.
24.	Singotamu et al., 2006 [58]	Asia	India	*P. falciparum*	Adults	No statistically significant differences in IL-2 between severe and non-severe malaria.
25.	Tatfeng and Agbonlahor 2008 [59]	Africa	Nigeria	*P. falciparum*	Not specified	No statistically significant differences in IL-2 between malaria and uninfected individuals.
26.	Tatfeng and Agbonlahor 2010 [60]	Africa	Nigeria	*P. falciparum*	Children and adults	IL-2 levels were significantly increased in malaria (children, adolescents, and adults) as compared to uninfected individuals.
27.	Tatfeng et al., 2012 [61]	Africa	Nigeria	*P. falciparum*	Children	IL-2 levels were significantly increased in patients with malaria as compared to uninfected individuals (all comparison groups).
28.	van den Bogaart et al., 2014 [62]	Africa	Sudan	*P. falciparum*	Children	No statistically significant differences in IL-2 between malaria and uninfected individuals.
29.	Wilson et al., 2010 [63]	Africa	Ghana	*P. falciparum*	Children and adults	No statistically significant differences in IL-2 between malaria and uninfected individuals.
30.	Zeyrek et al., 2006 [64]	Asia	Turkey	*P. vivax*	Children and adults	IL-2 levels were significantly increased in patients with malaria as compared to uninfected individuals.

Abbreviations: IL-2, interleukin-2

The meta-regression analysis suggested that continent was a confounding factor affecting the pooled effect estimate (*P* < 0.0001, Table S4). Subgroup analysis revealed that different continents and countries exhibited varying SMD (*P* < 0.0001, Table 3). For the different continents, studies conducted in Africa and Asia showed a similar trend, with IL-2 levels being higher in malaria patients compared to uninfected individuals. In contrast, a study conducted in South America showed no significant difference in IL-2 levels between the two groups (Table 3).

**Table 3:** Subgroup analyses of IL-2 levels between patients with malaria and uninfected individuals

Subgroup	Test for subgroup differences (random effects model)	SMD [95% CI]	*I*^2^ (%)	Number of studies
**Publication years**	0.08			
2010–2019		0.85 [0.14; 1.56]	92.9	3
2000–2009		12.59 [-10.79; 35.98]	99.7	2
Before 2000		0.00[-0.45; 0.45]	N/A	1
**Study design**	0.27			
Case-control studies		8.88 [-6.39; 24.15]	99.4	3
Cross-sectional studies		0.3443 [-0.01; 0.70]	55.2	3
**Continent**	< 0.0001			
Africa		0.73 [0.12; 1.34]	93.1	4
South America		0.35 [-0.09; 0.79]	N/A	1
Asia		24.56 [21.88; 27.23]	N/A	1
**Country**	< 0.0001			
Nigeria		1.11 [0.29; 1.93]	95.3	2
Ghana				
Democratic Republic of São Tomé and Príncipe		0.00 [-0.44; 0.45]	N/A	1
Malawi		0.62 [0.26; 0.98]	N/A	1
Brazil		0.3538 [-0.09; 0.79]	N/A	1
Turkey		24.56 [21.88; 27.23]	N/A	1
**Age ranges**	0.42			
Children		0.32 [-0.28; 0.93]	77.6	2
Children and adults		13.00 [-9.56; 35.57]	99.6	2
Not specified age group		0.57 [0.25; 0.90]	43.9	2
***Plasmodium*** **species**	0.33			
*P. falciparum*		0.72 [0.12; 1.34]	93.1	4
*P. vivax*		12.42 [-11.30; 36.14]	99.7	2
**Diagnostic method for malaria**	N/A			
Microscopy		4.5642 [-3.1598; 12.2881]	98.6	6
**Methods for IL-2**	0.25			
ELISA		12.24 [-11.82; 36.31]	99.7	2
Bead-based assay		0.5152 [0.2347; 0.7957]	0.0	2
ELISA/EIA		1.1148 [0.2979; 1.9316]	95.3	2
**Blood samples for IL-2**	0.24			
Serum		5.49 [-3.73; 14.72]	98.8	5
Plasma		0.00 [-0.44; 0.44]		1

Abbreviations: EIA, Enzyme immunoassay; ELISA, Enzyme-linked immunosorbent assay; RDT, rapid diagnostic test; CI, confidence interval; SMD, standardized mean difference; N/A, not assessed

### Qualitative and quantitative syntheses of IL-2 levels in patients with severe malaria and those with non-severe malaria

Among the 12 studies that investigated IL-2 levels in both patients with severe malaria and those with non-severe malaria, the majority (*n* = 9, 75%) reported no significant difference in IL-2 levels between the two groups [28, 41, 46, 48, 51, 52, 54, 56, 58]. Meanwhile, some studies (*n* = 2, 16.7%) reported increased IL-2 levels in severe malaria patients compared to those with non-severe malaria [26, 68]. One study found that IL-2 levels were not detectable in both severe and uncomplicated malaria cases (< 2 pg/ml) [53].

**Fig. 3:**
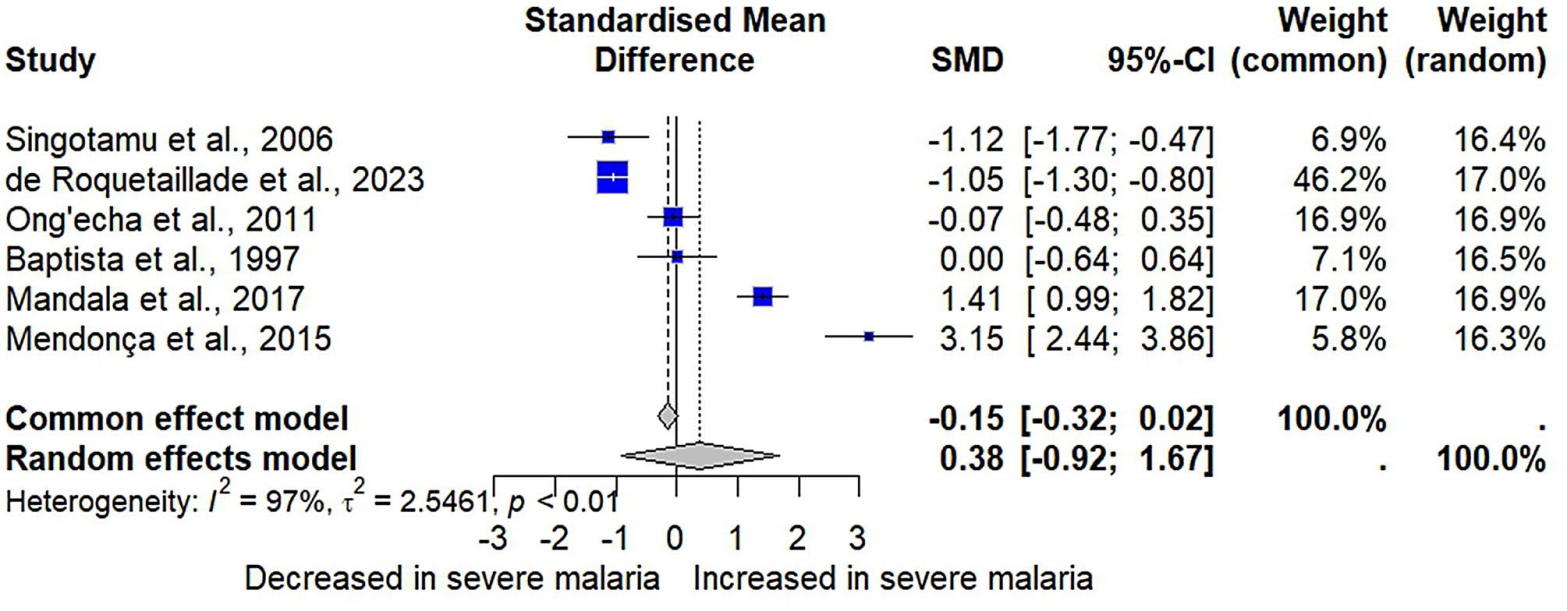
The forest plot compared IL-2 levels in patients with severe malaria compared to those with non-severe malaria. Explanations of the forest plot; blue square, effect estimate of individual study; gray diamond, pooled effect estimate; dash vertical line, pooled effect estimate; gray vertical line, no effect line; CI, confidence interval. SMD, standardized mean difference

Six studies provided data for a meta-analysis comparing IL-2 levels between patients with severe malaria (*n* = 328) and those with non-severe malaria (*n* = 366) [26, 28, 46, 49, 52, 58]. The results showed no significant difference in IL-2 levels between patients with severe and non-severe malaria (*P* = 0.57, SMD = 0.37, 95% CI [-0.91; 1.67], *I²* = 97.4%, 694 participants, random-effects model, Fig. 3). The fixed-effects model produced similar results (*P* = 0.08, SMD = -0.15, 95% CI [-0.32; 0.02], Fig. 3).

The meta-regression analysis suggested that continent, age range, and *Plasmodium* species were confounding factors affecting the pooled effect estimate (*P* < 0.05, Table S4). Subgroup analysis showed that publication year, study design, continent, country, age range, and *Plasmodium* species exhibited different SMD (*P* < 0.05, Table 4). For publication years, studies from 2020 to 2024 and 2000–2009 showed negative SMDs, indicating lower IL-2 levels in severe malaria, while studies from 2010 to 2019 showed no alteration in IL-2 levels between the two groups. In terms of study design, case-control studies showed lower IL-2 levels, while cross-sectional studies showed no significant difference between the two groups. Regarding continent and country, studies from South America reported increased IL-2 levels in severe malaria, while those from Asia and Europe reported decreased IL-2 levels in severe malaria. Age ranges showed decreased IL-2 levels in adults and increased IL-2 levels in studies that enrolled both children and adults. In terms of *Plasmodium* species, *P. falciparum* studies showed no difference in IL-2 levels between the two groups, whereas the *P. vivax* study showed increased IL-2 levels in patients with severe malaria. The method of IL-2 detection (ELISA vs. bead-based assay) and the blood sample type (plasma vs. serum) did not significantly affect the results.

**Table 4:** Subgroup analyses of IL-2 levels between patients with severe malaria and those with non-severe malaria

Subgroup	Test for subgroup differences (random effects model)	SMD [95% CI]	*I*^2^ (%)	Number of studies
**Publication years**	0.001			
2020–2024		-1.05 [-1.29; -0.79]	N/A	1
2010–2019		1.47 [-0.33; 3.28]	96.9	3
2000–2009		-1.12 [-1.77; -0.47]	N/A	1
Before 2000		0.00 [-0.64; 0.64]	N/A	1
**Study design**	0.02			
Cross-sectional study		0.67 [-0.76; 2.10]	96.2	5
Case-control study		-1.05 [-1.29; -0.79]	N/A	1
**Continent**	< 0.0001			
Africa		0.45 [-0.49; 1.41]	92.8	3
South America		3.15 [2.44; 3.86]	N/A	1
Asia		-1.12 [-1.77; -0.47]	N/A	1
Europe		-1.05 [-1.29; -0.79]	N/A	1
**Country**	< 0.0001			
Kenya		-0.07 [-0.48; 0.35]	N/A	1
Democratic Republic of São Tomé and Príncipe		0.00 [-0.64; 0.64]	N/A	1
Malawi		1.41 [0.99; 1.82]	N/A	1
Brazil		3.15 [2.44; 3.86]	N/A	1
India		-1.12 [-1.77; -0.47]	N/A	1
France		1.0477 [-1.29; -0.79]	N/A	1
**Age ranges**	< 0.0001			
Children		0.46 [-0.49; 1.41]	92.8	3
Adults		-1.06 [-1.29; -0.82]	N/A	2
Children and adults		3.15 [2.44; 3.86]	N/A	1
***Plasmodium*** **species**	< 0.0001			
*P. falciparum*		-0.16 [-1.07; 0.74]	96.3	5
*P. vivax*		3.15 [2.44; 3.86]	N/A	1
**Diagnostic method for malaria**	N/A			
Microscopy		0.38 [-0.92; 1.67]	97.4	6
**Methods for IL-2**	0.69			
ELISA		0.67 [-1.83; 3.17]	97.5	3
Bead-based assay		0.09 [-1.31; 1.48]	98.0	3
**Blood samples for IL-2**	0.12			
Plasma		0.17 [-1.34; 1.68]	96.9	5
Serum		1.40 [0.99; 1.82]	N/A	1

Abbreviations: EIA, Enzyme immunoassay; ELISA, Enzyme-linked immunosorbent assay; RDT, rapid diagnostic test; CI, confidence interval; SMD, standardized mean difference; N/A, not assessed

### Influential analysis

For the meta-analysis examining differences in IL-2 levels between patients with malaria and uninfected individuals, the influential analysis suggests that most individual studies do not dramatically change the overall outcome, except for Zeyrek et al. [64], which significantly impacts the meta-analysis results (Table S5.1). For the meta-analysis focusing on IL-2 levels in patients with severe versus non-severe malaria, the analysis shows that excluding any individual study does not meaningfully alter the overall conclusion: there is no significant difference in IL-2 levels between severe and non-severe malaria patients (Table S5.2).

### Publication bias

Publication bias analysis was not conducted because the number of studies included in the meta-analysis was below the recommended minimum of 10 [38].

### Discussion

The systematic review and meta-analysis provide a comprehensive synthesis of IL-2 levels in malaria patients compared to uninfected individuals, showing no significant difference between the two groups. However, variability was observed across factors such as continent and study location. Similarly, the analysis of IL-2 levels in patients with severe versus non-severe malaria also showed no significant difference, though variability was noted based on factors like publication year, study design, continent, country, age range, and *Plasmodium* species. The geographical variability in both analyses may be attributed to local disease epidemiology. Additionally, non-malarial febrile diseases in malaria-endemic areas [69] and genetic predispositions, such as IL-2 gene polymorphisms, could influence IL-2 levels [70].

Variability in publication years could stem from factors such as the use of different IL-2 quantification methods over time or geographic variations during the periods when the studies were conducted. Geographic differences (e.g., by continent) may also explain variations in IL-2 levels between *Plasmodium* species. For instance, the study by Zeyrek et al. [64] reported significantly higher median IL-2 levels in patients with *P. vivax* malaria compared to healthy controls. Excluding this study from the sensitivity analysis led to more stable meta-analysis results, suggesting that the immune response to *P. vivax* may differ from that of *P. falciparum.*

The IL-2 response to *Plasmodium* infection appears to be largely unaffected by age, aligning with previous research on age-related immune responses in malaria [30, 47, 52, 56, 71, 72]. One study even suggests that immunity levels are influenced not only by age and exposure but also by the cumulative number of clinical malaria episodes an individual has experienced [73]. This implies that cytokine responses, including IL-2 levels, in malaria patients may be affected by factors beyond age, such as the frequency of exposure to clinical malaria. Additionally, another study has shown that older children, compared to their younger counterparts at the same stage of infection, have reduced levels of pro-inflammatory cytokines like TNF-α, IL-2, and IL-6, as well as Th1-biased chemokines [74]. Supporting this complexity, a recent study found that areas with high transmission intensity exhibited decreased pro-inflammatory cytokine responses, including IL-2, during acute malaria, suggesting that repeated exposure may impact cytokine levels [75].

In addition to age, the clinical status of malaria may help explain the inconsistency in IL-2 levels between malaria patients and uninfected individuals. The interplay between cytokines could also influence this variation, as the immune response involves changes in cytokine levels. For instance, in severe malaria, elevated levels of IFN-γ and TNF-α, along with the upregulation of TGF-β mRNA, have been shown to cause downregulation of IL-12α and IL-2 levels, suggesting potential dysregulation of the cytokine network through T cell suppression [76]. A previous in vivo study indicated that IL-2 plays a role in balancing natural Treg cells and effector CD4(+) Th1 cells to eliminate *P. chabaudi* AS infection [77]. Another study found a significant association between IL-2 and IFN-γ levels [67], which is crucial for activating Natural Killer (NK) cells, leading to IFN-γ production—a critical cytokine for parasite control [78, 79]. However, it was observed that this interaction between IL-2 and IFN-γ weakens in the presence of *P. vivax* infection [67].

In general, IL-2 is essential for maintaining regulatory T cells (Tregs) and is a key factor in the differentiation of CD4 + T cells into specific effector subsets post-antigen activation. IL-2 also plays a critical role in promoting the expansion and differentiation of effector and memory cells within the CD8 + T cell compartment [80]. These functions underscore IL-2’s versatility and pivotal role in the immune response. Variations in IL-2 production can occur depending on the type of pathogen, the stage of infection, and the overall immune status of the host. Studies have highlighted IL-2’s significant role in malaria pathogenesis, as evidenced by elevated levels during *Plasmodium* infection [26, 47, 54, 61, 64, 66]. Given the unique immunological challenges posed by *Plasmodium* species, the role of IL-2 in malaria may differ from its function in other infections. For instance, in HIV infection, IL-2 deficiency occurs due to the dysfunction and destruction of CD4 + T cells [81]. In HIV patients, IL-2 administration can expand the CD4 + T-cell pool and help control viral replication [82]. In COVID-19 patients, IL-2 levels are elevated compared to healthy controls, contributing to severe inflammatory responses and cytokine storms [83, 84]. Moreover, IL-2 levels were significantly lower in COVID-19 non-survivors compared to survivors, indicating a worse prognosis for the disease [84]. In dengue infection, IL-2 levels were associated with dengue but did not significantly correlate with disease severity [85]. However, another study found that IL-2 levels were significantly elevated in severe dengue compared to non-severe cases [86]. In the present study, the meta-analysis did not find a significant correlation between IL-2 levels and malaria severity. These observations suggest that while IL-2 is critical in the immune response to malaria, dengue, and COVID-19 infections, its relationship with disease severity in malaria warrants further investigation through comprehensive studies.

There were limitations in the present study. Firstly, the high degree of heterogeneity in the data raises concerns about the stability of the pooled estimates. Secondly, the studies included in the meta-analysis employed various methods to quantify IL-2 levels, such as ELISA, bead-based assays, and radioimmunoassay. Thirdly, there were variations in the types of samples used for IL-2 detection, including serum and plasma. Although serum and plasma are the most typical biological samples for cytokine testing, cytokine levels in plasma have been identified as more stable than in serum samples [87]. Additionally, some of the included studies were cross-sectional in design, which limits the ability to assess temporal and causal relationships between IL-2 levels and malaria severity. Cross-sectional studies provide only a snapshot of cytokine levels at a single point in time, making it difficult to draw conclusions about the progression of immune responses throughout the infection. Finally, the limited number of studies included in the meta-analysis precluded an assessment of publication bias among the included studies. Future research should aim to expand on the present findings by incorporating a larger dataset of IL-2 measurements, providing a more comprehensive understanding of the role of IL-2 in malaria. Such studies are crucial for validating and extending our insights into the role of cytokines in malaria pathogenesis and could lead to more targeted diagnostic and therapeutic strategies.

## Conclusion

The systematic review and meta-analysis found no significant alteration in IL-2 levels in individuals with *Plasmodium* infections. Additionally, no significant difference in IL-2 levels was observed between individuals with different severities of malaria. Given the high variability in the analyses, further well-designed studies are needed to explore whether IL-2, in combination with other immunological biomarkers, could serve as a reliable indicator for *Plasmodium* infections and severe malaria.

## Supplementary Information

Below is the link to the electronic supplementary material.

Supplementary Material 1

Supplementary Material 2

Supplementary Material 3

Supplementary Material 4

Supplementary Material 5

## Data Availability

All relevant data are within the manuscript and its Supporting Information files.
