# Supplementary material for: Association between interleukin-2 cytokine levels and Plasmodium infections: a systematic review and meta-analysis
Source: BMC Infect Dis. 2025 Nov 5;25:1506. doi: 10.1186/s12879-025-11977-1 (PMC12587632; doi:10.1186/s12879-025-11977-1)
Supplement: Supplementary file 1 — Supplementary Material 1 [file 12879_2025_11977_MOESM1_ESM.docx]

**Association between interleukin-2 cytokine levels and *Plasmodium* infections: A systematic review and meta-analysis**

Pattamaporn Kwankaew^1^, Kwuntida Uthaisar Kotepui^1^, Nsoh Godwin Anabire^2,3^, Polrat Wilairatana^4*^, Manas Kotepui^1*^

^1^ Medical Technology, School of Allied Health Sciences, Walailak University, Tha Sala, Nakhon Si Thammarat, Thailand

^2^ Department of Biochemistry & Molecular Medicine, School of Medicine, University for Development studies, Tamale, Ghana.

^3^ West African Centre for Cell Biology of Infectious Pathogens (WACCBIP); Department of Biochemistry, Cell & Molecular Biology, University of Ghana, Accra, Ghana.

^4^ Department of Clinical Tropical Medicine, Faculty of Tropical Medicine, Mahidol University, Bangkok, Thailand

**^*^Corresponding author**

Manas Kotepui: manas.ko@wu.ac.th, Tel.: +66954392469

Polrat Wilairatana: [polrat.wil@mahidol.ac.th](mailto:polrat.wil@mahidol.ac.th)

Nsoh Godwin Anabire: nanabire@uds.edu.gh

Pattamaporn Kwankaew: pattamaporn.kw@wu.ac.th

Kwuntida Uthaisar Kotepui: [kwuntida.ut@wu.ac.th](mailto:kwuntida.ut@wu.ac.th)

**Table S1. Search terms**

**General keywords**

(“Interleukin 2” OR IL-2 OR IL2 OR TCGF OR “Lymphocyte Mitogenic Factor” OR “T-Cell Growth Factor” OR “T Cell Growth Factor” OR “T-Cell Stimulating Factor” OR “T Cell Stimulating Factor” OR “Thymocyte Stimulating Factor” OR “Interleukin II”) AND (malaria OR Plasmodiun OR “remittent fever” OR “marsh fever” OR paludism)

PubMed 3 September 2024

| No. | Key concept | Key concept | Results |
| --- | --- | --- | --- |
| 1. | IL-2 | interleukin-2[MeSH Terms] OR IL-2[MeSH Terms] OR “Interleukin 2”[Text Word] OR “Interleukin-2” [Text Word] OR IL-2[Text Word] OR IL2[Text Word] OR TCGF[Text Word] OR “Lymphocyte Mitogenic Factor” [Text Word] OR “T-Cell Growth Factor” [Text Word] OR “T Cell Growth Factor” [Text Word] OR “T-Cell Stimulating Factor” [Text Word] OR “T Cell Stimulating Factor”[Text Word] OR “Thymocyte Stimulating Factor”[Text Word] OR “Interleukin II”[Text Word] | 92,181 |
| 2. | Malaria | malaria[MeSH Terms] OR Plasmodium[MeSH Terms] OR malaria[Text Word] OR Plasmodium[Text Word] OR “remittent fever”[Text Word] OR “marsh fever”[Text Word] OR paludism[Text Word] | 123,543 |
| 3. | #1 AND #2 | #1 AND #2 | 476 |

Embase 3 September 2024

| No. | Key concept | Search terms | Results |
| --- | --- | --- | --- |
| 1. | IL-2 | 'interleukin 2'/exp OR 'interleukin 2':ti,ab,kw,de OR 'interleukin-2'/exp OR 'interleukin-2':ti,ab,kw,de OR 'il2'/exp OR 'il 2':ti,ab,kw,de OR il2:ti,ab,kw,de OR tcgf OR 'interleukin 2':ti,ab,kw,de OR 'lymphocyte mitogenic factor'/exp OR 'lymphocyte mitogenic factor':ti,ab,kw,de OR 't-cell growth factor'/exp OR 't-cell growth factor':ti,ab,kw,de OR 't cell growth factor'/exp OR 't cell growth factor':ti,ab,kw,de OR 't-cell stimulating factor':ti,ab,kw,de OR 't cell stimulating factor':ti,ab,kw,de OR 'thymocyte stimulating factor':ti,ab,kw,de OR 'interleukin ii'/exp OR 'interleukin ii':ti,ab,kw,de | 180,856 |
| 2. | Malaria | malaria/exp OR plasmodium/exp OR 'remittent fever'/exp OR 'marsh fever'/exp OR paludism/exp OR malaria:ti,ab,kw,de OR plasmodium:ti,ab,kw,de OR 'remittent fever':ti,ab,kw,de OR 'marsh fever':ti,ab,kw,de OR paludism:ti,ab,kw,de | 163,748 |
| 3. | #1 AND #2 | ('interleukin 2'/exp OR 'interleukin 2':ti,ab,kw,de OR 'interleukin-2'/exp OR 'interleukin-2':ti,ab,kw,de OR 'il2'/exp OR 'il 2':ti,ab,kw,de OR il2:ti,ab,kw,de OR tcgf OR 'interleukin 2':ti,ab,kw,de OR 'lymphocyte mitogenic factor'/exp OR 'lymphocyte mitogenic factor':ti,ab,kw,de OR 't-cell growth factor'/exp OR 't-cell growth factor':ti,ab,kw,de OR 't cell growth factor'/exp OR 't cell growth factor':ti,ab,kw,de OR 't-cell stimulating factor':ti,ab,kw,de OR 't cell stimulating factor':ti,ab,kw,de OR 'thymocyte stimulating factor':ti,ab,kw,de OR 'interleukin ii'/exp OR 'interleukin ii':ti,ab,kw,de) AND (malaria/exp OR plasmodium/exp OR 'remittent fever'/exp OR 'marsh fever'/exp OR paludism/exp OR malaria:ti,ab,kw,de OR plasmodium:ti,ab,kw,de OR 'remittent fever':ti,ab,kw,de OR 'marsh fever':ti,ab,kw,de OR paludism:ti,ab,kw,de) | 1,104 |

Scopus 3 September 2024

| No. | Key concept | Search terms | Results |
| --- | --- | --- | --- |
| 1. | IL-2 | TITLE-ABS-KEY (“Interleukin 2” OR “Interleukin-2” OR IL-2 OR IL2 OR TCGF OR “Interleukin 2” OR “Lymphocyte Mitogenic Factor” OR “T-Cell Growth Factor” OR “T Cell Growth Factor” OR “T-Cell Stimulating Factor” OR “T Cell Stimulating Factor” OR “Thymocyte Stimulating Factor” OR “Interleukin II”) | 162,356 |
| 2. | Malaria | malaria OR Plasmodiun OR “remittent fever” OR “marsh fever” OR paludism | 147,241 |
| 3. | 1 AND 2 | TITLE-ABS-KEY (“Interleukin 2” OR “Interleukin-2” OR IL-2 OR IL2 OR TCGF OR “Interleukin 2” OR “Lymphocyte Mitogenic Factor” OR “T-Cell Growth Factor” OR “T Cell Growth Factor” OR “T-Cell Stimulating Factor” OR “T Cell Stimulating Factor” OR “Thymocyte Stimulating Factor” OR “Interleukin II”) AND (malaria OR Plasmodiun OR “remittent fever” OR “marsh fever” OR paludism) | 934 |

MEDLINE 3 September 2024

| No. | Key concept | Search terms | Results |
| --- | --- | --- | --- |
| 1. | IL-2 AND Malaria | (“Interleukin 2” OR “Interleukin-2” OR IL-2 OR IL2 OR TCGF OR “Interleukin 2” OR “Lymphocyte Mitogenic Factor” OR “T-Cell Growth Factor” OR “T Cell Growth Factor” OR “T-Cell Stimulating Factor” OR “T Cell Stimulating Factor” OR “Thymocyte Stimulating Factor” OR “Interleukin II”) AND (malaria OR Plasmodium OR “remittent fever” OR “marsh fever” OR paludism) | 482 |

CENTRAL 3 September 2024

| No. | Key concept | Search terms | Results |
| --- | --- | --- | --- |
| 1. | IL-2 AND Malaria | (“Interleukin 2” OR “Interleukin-2” OR IL-2 OR IL2 OR TCGF OR “Interleukin 2” OR “Lymphocyte Mitogenic Factor” OR “T-Cell Growth Factor” OR “T Cell Growth Factor” OR “T-Cell Stimulating Factor” OR “T Cell Stimulating Factor” OR “Thymocyte Stimulating Factor” OR “Interleukin II”) AND (malaria OR Plasmodium OR “remittent fever” OR “marsh fever” OR paludism) in Title Abstract Keyword | 29 |
