# Supplementary material for: Association between interleukin-2 cytokine levels and Plasmodium infections: a systematic review and meta-analysis
Source: BMC Infect Dis. 2025 Nov 5;25:1506. doi: 10.1186/s12879-025-11977-1 (PMC12587632; doi:10.1186/s12879-025-11977-1)
Supplement: Supplementary file 4 — Supplementary Material 4 [file 12879_2025_11977_MOESM4_ESM.docx]

**Table S4. Meta-regression analyses**

**Table S4.1 Meta-regression analysis of IL-2 in malaria patients vs. non-malarial individuals.**

| **Covariates** | **tau^2^** | ***I^2^* (%)** | **R-squared (%)** | **Test for Residual Heterogeneity, *P* value** | **Test of Moderators, *P* value** |
| --- | --- | --- | --- | --- | --- |
| Publication years | 93.62 | 99.95 | 0.00 | < 0.0001 | 0.36 |
| Study design | 89.60 | 99.96 | 3.50 | < 0.0001 | 0.27 |
| Continent | 0.36 | 93.52 | 99.61 | < 0.0001 | < 0.0001 |
| Age group | 86.94 | 99.94 | 6.36 | < 0.0001 | 0.30 |
| *Plasmodium* species | 71.90 | 99.95 | 22.55 | < 0.0001 | 0.11 |
| Diagnostic method for malaria | N/A | N/A | N/A | N/A | N/A |
| Method for IL-2 measurement | 99.10 | 99.96 | 0.00 | < 0.0001 | 0.42 |
| Blood samples for IL-2 measurement | 110.51 | 99.97 | 0.00 | < 0.0001 | 0.63 |

N/A, not assessed.

**Table S4.2 Meta-regression analysis of IL-2 in severe malaria vs. non- severe malaria patients.**

| **Covariates** | **tau^2^** | ***I^2^* (%)** | **R-squared (%)** | **Test for Residual Heterogeneity, *P* value** | **Test of Moderators, *P* value** |
| --- | --- | --- | --- | --- | --- |
| Publication years | 2.48 | 97.56 | 2.28 | < 0.0001 | 0.36 |
| Study design | 2.58 | 97.19 | 0.00 | < 0.0001 | 0.33 |
| Continent | 0.65 | 91.72 | 74.47 | < 0.0001 | 0.001 |
| Age group | 0.42 | 87.50 | 83.50 | < 0.0001 | < 0.0001 |
| *Plasmodium* species | 1.00 | 95.53 | 60.45 | < 0.0001 | 0.004 |
| Diagnostic method for malaria | N/A | N/A | N/A | N/A | N/A |
| Method for IL-2 measurement | 3.08 | 98.38 | 0.00 | < 0.0001 | 0.69 |
| Blood samples for IL-2 measurement | 2.88 | 98.03 | 0.00 | < 0.0001 | 0.51 |

N/A, not assessed.
