# Supplementary material for: Association between interleukin-2 cytokine levels and Plasmodium infections: a systematic review and meta-analysis
Source: BMC Infect Dis. 2025 Nov 5;25:1506. doi: 10.1186/s12879-025-11977-1 (PMC12587632; doi:10.1186/s12879-025-11977-1)
Supplement: Supplementary file 5 — Supplementary Material 5 [file 12879_2025_11977_MOESM5_ESM.docx]

**Table S5. Influential analyses**

**Table S5.1 Influential analyses of IL-2 in malaria patients vs. non-malarial individuals.**

Influential analysis (random effects model)

SMD 95%-CI p-value tau^2 tau

Omitting Baptista et al., 1997 5.4920 [-3.7388; 14.7228] 0.2436 110.5138 10.5126

Omitting Mandala et al., 2017 5.3684 [-3.9295; 14.6663] 0.2578 112.1279 10.5890

Omitting Scherer et al., 2016 5.4215 [-3.8486; 14.6917] 0.2517 111.4613 10.5575

Omitting Tatfeng and Agbonlahor 2008 5.3530 [-3.9528; 14.6588] 0.2596 112.3159 10.5979

Omitting Tatfeng and Agbonlahor 2010 5.1866 [-4.1911; 14.5644] 0.2784 114.0667 10.6802

Omitting Zeyrek et al., 2006 0.6612 [ 0.1580; 1.1644] 0.0100 0.2962 0.5442

Pooled estimate 4.5642 [-3.1598; 12.2881] 0.2468 92.8486 9.6358

I^2

Omitting Baptista et al., 1997 98.8%

Omitting Mandala et al., 2017 98.8%

Omitting Scherer et al., 2016 98.8%

Omitting Tatfeng and Agbonlahor 2008 98.8%

Omitting Tatfeng and Agbonlahor 2010 98.7%

Omitting Zeyrek et al., 2006 91.7%

Pooled estimate 98.6%

Details on meta-analytical method:

- Inverse variance method

- Restricted maximum-likelihood estimator for tau^2

**Table S5.2 Influential analyses of IL-2 in severe malaria vs. non- severe malaria patients.**

Influential analysis (random effects model)

SMD 95%-CI p-value tau^2 tau I^2

Omitting Baptista et al., 1997 0.4538 [-1.1228; 2.0305] 0.5726 3.1667 1.7795 98.0%

Omitting de Roquetaillade et al., 2023 0.6698 [-0.7616; 2.1012] 0.3591 2.5801 1.6063 96.2%

Omitting Mandala et al., 2017 0.1701 [-1.3392; 1.6795] 0.8251 2.8845 1.6984 96.9%

Omitting Mendonça et al., 2015 -0.1613 [-1.0679; 0.7453] 0.7273 1.0069 1.0034 96.3%

Omitting Ong'echa et al., 2011 0.4691 [-1.1090; 2.0471] 0.5602 3.1604 1.7778 98.0%

Omitting Singotamu et al., 2006 0.6718 [-0.7469; 2.0905] 0.3534 2.5519 1.5975 97.9%

Pooled estimate 0.3770 [-0.9183; 1.6724] 0.5684 2.5461 1.5956 97.4%

Details on meta-analytical method:

- Inverse variance method

- Restricted maximum-likelihood estimator for tau^2
